# Barriers and facilitators to the uptake of the Concussion Awareness Training Tool as continuing medical education in primary care

**DOI:** 10.2217/cnc-2022-0014

**Published:** 2023-05-19

**Authors:** Jalila Devji, Shazya Karmali, Kate Turcotte, Shelina Babul

**Affiliations:** 1 Faculty of Medicine Undergraduate Program, University of British Columbia, Vancouver, V6T 1Z3, Canada; 2 BC Injury Research and Prevention Unit, BC Children's Hospital, Vancouver, V6H 3V4, Canada; 3 Department of Pediatrics, Faculty of Medicine, University of British Columbia, Vancouver, V6T 1Z3, Canada

**Keywords:** concussion, mild TBI, sports medicine, treatment

## Abstract

**Aim:**

Continuing medical education (CME) informs physicians on current research. The Concussion Awareness Training Tool (CATT) provides education on concussion diagnosis and treatment. The aims of this study were to explore physician CME practices and preferences, understand barriers and facilitators to implementing the CATT as CME, and provide recommendations.

**Materials & methods:**

Physicians in British Columbia, Canada participated in an online survey and telephone interview. Descriptive analysis of quantitative data, and text-based data analysis were undertaken to identify themes.

**Results:**

Barriers included lack of time and awareness of the resource. Facilitators were its ease of use, accessibility, conciseness and comprehensiveness.

**Conclusion:**

The perceptions of barriers and facilitators reported by physicians are important to understand and better promote the use of the CATT.

## Plain language summary

Doctors take continuing medical education (CME) to stay on top of new information and practices. The Concussion Awareness Training Tool (CATT) is available for doctors to learn more about concussion care. This study looked at how doctors in British Columbia, Canada are taking CME training, and what factors are encouraging or preventing them from using the CATT. Doctors reported a lack of time for taking CME, and not knowing about this resource. Those who had taken the CATT training reported that it is easy to use, accessible, concise, and comprehensive. This information will be used to improve promotion of this resource.

## Tweetable abstract

Identifying barriers and facilitators to implementing concussion education for continuing medical education will inform optimal ways to reach physicians; the Concussion Awareness Training Tool is evidence-based, accessible, concise, comprehensive, and free.

Concussion education for healthcare providers is a public health priority [1]. Concussions accounted for 9.7% of the 42,766 head injury hospitalizations in British Columbia, Canada from 2001 to 2010, with the majority of cases treated in emergency departments, doctors offices, walk-in clinics or not at all [2]. Primary care providers, including pediatricians, family and emergency medicine physicians, and nurse practitioners are vital in the recognition, assessment, and prevention of concussions [3]. However, concussion diagnosis and management recommendations are not consistently translated into medical practice [4].

The Concussion Awareness Training Tool (CATT), originally launched in 2013, is a comprehensive online tool with the aim of supporting concussion recognition, diagnosis, treatment, and management in Canada. The 2013 pre/post-intervention evaluation found a statistically significant positive change in physician practices following completion of the CATT (p < 0.01) [5]. A more recent quality assessment/quality improvement study of the CATT found that the majority of physician respondents reported the following benefits from using the tool: learning new information (85%), changing the way they diagnose, treat or manage concussion (73%); and recommending CATT to colleagues (71%) [6]. The tool consists of eight online concussion education modules, each tailored to a specific audience. The resource is evidence-based, free of charge, and provides medical professionals with flexibility in navigating and reviewing course content. Importantly, the CATT is updated regularly to incorporate new research and resources, and includes patient resources for physicians to use as handouts.

Earlier research has identified several barriers to the successful implementation of continuing medical education (CME). Commonly identified barriers include lack of time due to excessive clinical and administrative responsibilities [7–9], increased workload due to time away from practice to engage in CME [10], and an inability to obtain clinical coverage while engaging in CME [10,11]. Another barrier to accessing CME is geographical isolation, for instance distance and travel time to main education centers and CME programs from remote locations [7–11]. Characteristics of CME activities identified as obstacles to participation included lack of high-quality activities, courses that run for a year or longer and information overload [10,11]. Other identified barriers include lack of physician motivation, personal and family commitments and fatigue [7–10]. Additional challenges that have been reported in relation to the use of technology for CME include an excess of information to scan, an inability to find information, low digital competence, technical issues, and inadequate searching skills [12,13].

Understanding barriers to the completion of online CME for general practitioners is of utmost importance in ensuring its successful implementation [14]. There is limited evidence regarding barriers to the adoption of the CATT as a form of CME to promote appropriate concussion management in primary care in British Columbia. The aims of this study were to: understand physicians’ current CME practices and preferences; understand barriers and facilitators to implementing the CATT as a form of CME; and to provide recommendations to improve the uptake of the CATT.

## Materials & methods

### Study protocol

The study received approval from the University of British Columbia Children's & Women’s Hospital Research Ethics Board (H20-01546). The study population included practicing primary healthcare physicians/general practitioners in British Columbia. A list of 52 cities was generated. Two cities were selected (Maple Ridge and Pitt Meadows) to test the methodology in an area of familiarity. The remaining 50 cities were randomized using Excel, and physicians in the first 29 cities of the list were contacted. E-mail addresses of prospective respondents were obtained by telephone, using the Registrant Directory from the College of Physicians and Surgeons of British Columbia website. Multi physician offices were targeted, and approximately half of single physician offices (comprising 20% of the list) were also contacted. A link to the study survey was emailed to each physician for whom an email address was obtained.

In addition, the BC Injury Research and Prevention Unit, Doctors of BC, the University of British Columbia Faculty of Medicine and the BC College of Family Physicians distributed the link to the study survey through their newsletters. An incentive – a draw for an Apple Watch – was later introduced to increase response rate. Respondents who completed the online survey were invited to participate in a follow-up telephone interview.

### Survey

A survey was developed and validated using face validation. Selected representatives from the target audience were asked to describe how long it took them to complete the survey, whether they felt the survey addressed the research objectives, and if they had any suggested changes or edits.

The survey was administered through REDCap, a secure web application used by researchers to collect and store data. Physicians were invited to complete the survey and all data were deidentified. The survey included multiple choice and short answer questions related to CME, concussion knowledge, concussion assessment, concussion management, and the CATT specifically (Supplementary Materials). For some questions, participants had the option of selecting all responses that applied to them. If respondents agreed, they were contacted by researchers for a follow-up interview. Interviews were held over the phone and audio recorded.

### Data analysis

Descriptive statistics of quantitative data were completed using Excel, including counts and proportions. Interviews were deidentified and transcribed verbatim. Qualitative data, from both the survey and transcribed from follow-up interviews, were analyzed with NVivo 12 using inductive content analysis, which allows for themes to emerge from question responses (i.e., themes were not predetermined) [15]. Two researchers independently reviewed responses, identified themes and then met to review, compare and sort information. Any discrepancies were discussed until consensus on final themes and subthemes was reached.

## Results

### Survey validation

Thirteen surveys were distributed by email for validation, of which 12 were returned with responses. In total, 41.7% (*n* = 5) of participants indicated that the survey took 15 min to complete, with a third of participants (*n* = 4) reporting a shorter time period and one quarter reporting a longer period (*n* = 3). The majority (83.3%, *n* = 10) of participants indicated they felt the survey addressed the research objectives, while the remainder (*n* = 2) felt the survey somewhat addressed the objectives. Four participants (33.3%) provided suggested changes for the survey to better address research objectives; one participant’s suggestion was incorporated into the final survey.

### Survey response rate & respondent demographics

A total of 658 email invitations were distributed and 43 responses were received. An additional five responses were received from survey links distributed through newsletters, for a total of 48 responses. Of these, 29 surveys were complete (operationalized as having completed the final question of the survey), 13 were partially complete (i.e., at least one question answered) and six were incomplete (i.e., consent not given and/or no questions answered). The six incomplete surveys were excluded from the study, as well as one respondent who did not meet the inclusion criteria, for a total of 41 surveys included in the analysis. The final response rate was 6.2% (41/658). Fifteen respondents agreed to the follow-up telephone interview; however, eight interviews were conducted due to loss to follow-up.

Over two-thirds of respondents (n = 28) completed the demographic questions (Table 1). All respondents were identified as practicing family medicine physicians/general practitioners providing direct patient/clinical care. Years practicing medicine ranged from less than 6 years to over 30 years, 68% were working full-time, 68% served an urban/suburban population, 82% worked at a private office or clinic and 54% reported working 50 h or more per week.

**Table 1.** Respondents’ demographics.

Demographic	Proportion, % (n)
Years practicing medicine:	
– <6 years	17.9 (5)
– 6–10 years	17.9 (5)
– 11–20 years	14.3 (4)
– 21–30 years	17.9 (5)
– >3 years	32.1 (9)
Location of medical school:	
– British Columbia	28.6 (8)
– Alberta	21.4 (6)
– Ontario	21.4 (6)
– Manitoba	3.6 (1)
– Outside Canada	25.0 (7)
Current employment:	
– Part time	32.1 (9)
– Full time	67.9 (19)
Population:	
– Urban/suburban	67.9 (19)
– Rural	28.6 (8)
– Other^†^	3.6 (1)
Primary work setting:	
– Private office/clinic	82.1 (23)
– Hospital	3.6 (1)
– Other^‡^	14.3 (4)
Average work week:	
– <15 h	10.7 (3)
– 30–39 h	25.0 (7)
– 40–49 h	10.7 (3)
– 50–59 h	39.3 (11)
– 60+ h	14.3 (4)

† Regional.

‡ Long-term care facilities; 50/50 hospital (including emergency room) and clinic; hospice; full scope rural family practice, working mix of private clinic, emergency, hospital and maternity care.

### Physicians’ current CME practices & preferences

When asked about the number of hours per week dedicated to CME/professional development, 31.6% of respondents (*n* = 13) reported dedicating up to 1.5 h per week, 53.7% (n = 22) reported dedicating 2–3 h per week and 14.5% (*n* = 6) reported dedicating 5 h or more.

The majority of respondents (75.6%, n = 31) indicated that they would most likely engage in online CME/professional development, whereas 17.1% (n = 7) were more likely to take part in in-person CME/professional development, and 7.3% (n = 3) in other forms, such as print, books/journals, and/or podcasts. When asked which CME/professional development format they prefer most, 48.8% of respondents (n = 20) indicated in-person, 46.3% (n = 19) online and 4.9% (n = 2) selected other (e.g., journal articles or podcasts).

Respondents were asked where and how often they receive updated information on concussion recognition, diagnosis, treatment, and management. Of the 41 survey respondents, 32 provided further information through the survey and eight through the follow-up interview, for a total of 40 respondents. Twenty-five (62.5%) noted that they do not receive updated concussion information often, five (12.5%) seek information as needed for specific patients, eight (20.0%) receive information one to three times per year, and five (12.5%) receive updated concussion information regularly (monthly or more frequently). Sixteen respondents (40.0%) noted that they most frequently refer to online resources for concussion information, with many identifying *UptoDate* – an electronic clinical resource tool for physicians and patients – as a key online resource for concussion information. Fourteen (35.0%) respondents reference journals for concussion CME, while 10 (25.0%) refer to external organizations’ resources (e.g., Canadian Academy of Sport and Exercise Medicine – CAESM, Parachute – the national NGO for injury prevention, the CATT, and Concussions Ontario). Conferences, events, webinars, and journal clubs were also noted as sources of current concussion information by six respondents (15.0%) and four respondents (10.0%) consult other professionals in the field. Current physician CME practices and preferences are summarized in Table 2.

**Table 2.** Summary of physicians’ current continuing medical education practices and preferences.

Current CME practices/preferences	Proportion, % (n)
Number of hours per week dedicated to CME/professional development:	
– 1.5 h	31.6 (13)
– 2–3 h	53.7 (22)
– 5 h or more	14.5 (6)
Format of CME/professional development most likely to engage in:	
– Online	75.6 (31)
– In-person	17.1 (7)
– Other	7.3 (3)
Preferred format of CME/professional development:	
– In-person	48.8 (20)
– Online	46.3 (19)
– Other	4.9 (2)
Frequency of updated information sought on concussion care:	
– Not often	62.5 (25)
– For specific patients	12.5 (5)
– One- to three-times per year	20.0 (8)
– Regularly (monthly or more)	12.5 (5)
Format of resources used to seek updated concussion information:	
– Online	40.0 (16)
– Journals	35.0 (14)
– External organizations	25.0 (10)
– Other professionals in the field	10.0 (4)
– Other	15.0 (6)

CME: Continuing medical education.

### Barriers & facilitators to implementing CATT as a form of CME

Survey results indicated that 30.3% (*n* = 10) of respondents were aware of the CATT. Eight survey respondents and two who engaged in a follow-up interview further described that they learned about the CATT from education sessions/sources and conferences (*n* = 3), external organizations (*n* = 3) and online (*n* = 2). Two were unsure where they learned about the CATT. Of the three respondents who had previously used the CATT, two reported that they visit the site monthly and one that they visit the site three-times per month on average. All three of these respondents noted that the CATT has had an impact on their concussion recognition, diagnosis, treatment, and management; one noted that the CATT supports their concussion practices as it contains all information in one place, while another explained that it facilitates patient education.

Twenty-eight (68.3%) survey respondents and eight interviewees provided the following responses when asked about factors that might facilitate/encourage them to use the CATT as a source for CME (Table 3).

**Table 3.** Summary of facilitators and barriers to the use of the Concussion Awareness Training Tool.

Use of the CATT	Proportion, % (n)
Facilitators to the use of the CATT	
Ease of use, straightforwardness and conciseness	38.9 (14)
Additional handouts and guidelines	8.3 (3)

**Barriers to the use of the CATT**	
Lack of time	52.8 (19)
Not easy to access or use	22.2 (8)
Lack of awareness	16.7 (6)
Irrelevance to practice	8.3 (3)

CATT: Concussion Awareness Training Tool.

Characteristics of the CATT such as ease of use, accessibility, straightforwardness, and conciseness were noted as key factors to encourage the use of the CATT (n = 14). One respondent stated that the CATT provides “clear and concise patient information sheets [regarding concussion] management and what to do, [and] contains easy to use/print symptom assessment tools”. Three respondents noted that the additional handouts and guidelines for doctors and patients provided on the CATT site are reasons they continue to use the resource. One respondent described “One of the things that I thought was very, very useful is from the medical professional point of view is you guys had really good handouts which are really nice to give to either the individual or whoever comes with them…So, I actually kept them open on my computer, there’s one about…understanding your concussion, and what to expect, and red flags and those types of things…and those standardized sheets that can be given to school or workplace”.

Additional suggestions to facilitate the uptake of CATT included: increasing awareness of the resource (n = 6); incorporating it into CME credits or events (n = 4); ensuring that the CATT is evidence-based and current (n = 3); increasing availability of the CATT on other sites used frequently by physicians (n = 1); containing information for many different audiences (n = 1); and, the incentive of compensation (n = 1). One respondent noted that the number of their patients suffering from concussions would dictate their use of the CATT.

Twenty-eight (68.3%) survey respondents and eight interviewees (36 in total) also identified barriers that might discourage them from using the CATT for CME. Lack of time during an office examination (52.8%, n = 19) was a key barrier to using CATT resources. One respondent described the difficulty of adding additional assessments to their current concussion evaluations which already include comprehensive clinical tools (e.g., SCAT-5, Rivermead Post-Concussion Symptom Questionnaire): “I think because the evaluations are very extensive and detailed… the challenge for me would be trying to get all of it completed”. Eight respondents (22.2%) noted that a barrier would be present if the clinical tools are not easy to access or use. One respondent suggested creating a CATT smartphone application. Not enough awareness of CATT (16.7%, n = 6), and irrelevance to practice (8.3%, n = 3) were also cited as barriers to use.

Suggestions to increase the CATT’s reach to physicians, as provided by the eight respondents who completed the follow-up interview, included engaging with the Divisions of Family Practice, CME, Doctors of BC, and Pathways online medical care and community services directory, as well as utilizing various engagement strategies, such as journals, email and word of mouth.

## Discussion

A growing number of patients are now seeking concussion care, as concussion continues to gain recognition as an important public health issue [16]; however, medical professionals have noted a lack of resources for optimal concussion management [6,17]. Given that optimal concussion management can have a significant impact on a patient’s quality of life, accurate and up-to-date concussion training and resources are essential for all medical professionals, and for family medicine physicians/general practitioners in particular [16]. The CATT for Medical Professionals is an important resource for evidence-based approaches to concussion care [6]. Thus, the purpose of this research was to understand physicians' current CME practices and preferences; understand barriers and facilitators to implementing the CATT as a form of CME; and to provide recommendations to improve the uptake of the CATT.

It is clear from the current study and previous research that physicians have indicated a preference for online CME [4]. The CATT is ideally suited to disseminate concussion education given the increased prevalence of computer use and internet access in the medical field. In addition, the current medical climate in the context of the COVID-19 pandemic exemplifies that the online delivery of the CATT modules is a convenient and easily accessible format for education delivery and self-directed learning. O'Brien and colleagues (2021) investigated factors that influence clinicians in selecting CME activities in the USA, and found that the feature of lowest appeal in an online course was also the feature of greatest appeal in an in-person course, in that the respondent simply showed a preference for the modality in which one learned better [18]. This suggests that there may always be a discrepancy between preference for online versus in-person learning, given that it will depend on the subjective experience of the individual.

Respondents in this study outlined barriers (e.g., lack of time) that would prevent them from using the CATT, which are common barriers to engaging in CME that have been reported by other researchers [18–20]. Importantly, Reis and colleagues (2022) also outlined additional considerations related to CME for physicians, in that some have reported that they feel their training is insufficient in relation to the variety of clinical situations they encounter on a daily basis, especially considering they work on the medical frontlines [20]. As a result, they require ongoing, up-to-date training in many areas [20]. When compounded with their lack of time, they have reported negative feelings associated with engaging in CME, such as a lack of discipline, laziness, guilt, and saturation of work [20]. These are important factors to consider when engaging with physicians and tailoring CME to suit their needs and routines. Given that many physicians in British Columbia have reported time constraints when attempting to conduct comprehensive patient assessments, and lack of standardized concussion assessments, the Government of British Columbia has a new 2022 Guidelines and Protocols Advisory Committee (GPAC) currently working to develop guidelines for general practitioners to assess and manage concussions, which are expected to be available in 2023. The goal is for these guidelines is to streamline concussion assessments and lay out an assessment and management plan through for in-office appointments.

When asked to describe facilitators and uptake of the CATT, some respondents in the current study suggested developing a smartphone application (‘app’) to support in-office clinical decision making, improve accuracy and efficiency and enhance productivity. While the CATT program receives base funding from the British Columbia government for sustainability, as a free online evidence-based resource, soliciting funding for the development of a clinical assessment app has not been feasible. However, the CATT for Medical Professionals module includes a collection of clinical guidelines, assessments and additional relevant resources (e.g., guidelines for diagnosing and managing pediatric concussion). These resources, as well as an online version of the SCAT5 and Child SCAT5, are easily accessible and free of charge on the CATT website via smartphone, tablet, and computer. It has been reported that important facilitators to self-directed learning for physicians are the tools and resources provided by the programs they are engaging in. Physicians have reported that being directed to structured tools to appraise relevance of information to their practice, handout materials, and resources and tools that acted as a catalyst to encourage reflection on one’s current practice were facilitators to engaging in self-directed learning programs [21]. In a USA based survey, physicians, nurse practitioners, and physician assistants reported that online courses for CME allow them to engage in content at their own pace and within their own schedules [18]. Researchers have evaluated other forms of online CME and reported that methods such as massive online open courses and webinar-based learning are also effective methods for engaging physicians in virtual learning [22,23]. For instance, physicians who engaged in webinar-based CME in otorhinolaryngology during the COVID-19 pandemic were surveyed regarding their participation, and of the 780 participants who attended all 20 webinars, 68% indicated preference for online training versus in-person [23].

Based on results from the current study, nearly 70% of physicians noted that they spent at least 2 hours per week on CME. A maximum of 2 hours can be claimed for study credits for CME for physicians as an Accredited Self-Assessment Program as defined by the Maintenance of Certification Program of the Royal College of Physicians and Surgeons of Canada. Thus, given that the CATT for Medical Professionals module takes an estimated 2 hours to complete, it fits well as a CME accredited self-assessment program. Moreover, many of the features that physicians indicated would be important components of CME programs are existing components of the CATT, such as ease of use, ease of access, conciseness, evidence-based, and up-to-date. Additional resources are also available for doctors and patients, including information for many different audiences, which physicians noted as key facilitators to the use of the CATT. At last, some respondents suggested monetary compensation for physicians to complete the CATT training, in order to increase its use. However, the CATT is a free resource currently dependent on government funding for its sustainability, therefore this suggestion is not feasible.

Recommendations to address the barriers and promote the facilitators to the use of the CATT included the incorporation of the CATT into Canadian medical schools, and a focused communications strategy. As of 2021/2022, concussion has been incorporated into the University of British Columbia medical school curriculum as MEDD 448: Concussion: Diagnosis and Management, a 90 min lecture and dedicated time to complete the CATT training. Continued efforts to are planned to connect with other medical schools in Canada, to advocate for the inclusion of concussion training prior to entry into clinical practice.

Recommendations for a focused communication strategy for the CATT include where to advertise and what to highlight in the advertisement. Suggestions for a tailored and targeted communication strategy include the following methods. First, sustainable connections continue to be built with provincial and national medical associations (e.g., Divisions of Family Practice, Doctors of BC). Agreements with these organizations to distribute reminders to their membership would be beneficial. Another method to increase CATT uptake would be to make it accessible via key resources utilized by physicians, such as UpToDate or DynaMed, and advertising in journals commonly accessed by physicians (e.g., BC Medical Journal or Canadian Medical Association Journal), as well as on their websites. This will require the allocation of funding for advertisements and promotion. At last, although many CATT resources appear on the Pathways website already, including a direct link to the CATT home page will be a streamlined method to ensure that physicians have access to the website, and available resources, as a whole. The CATT website provides information for diverse, targeted audiences. In addition to medical professionals, there are seven other online educational modules targeted to coaches, parents and caregivers, school professionals, youth (high school), high performance athletes (university/college), workers and workplaces and women’s support workers supporting survivors of intimate partner violence.

## Limitations

While efforts were made to engage with physicians through channels they would typically engage with (e.g., Doctors of BC, the University of British Columbia Faculty of Medicine, BC College of Family Physicians and the BC Injury Research and Prevention Unit newsletters), survey uptake and completion were low. Nonresponse bias is inherent in survey dissemination, in that participants may either choose not to participate or decide not to complete a survey after starting it. Low response rate could have been influenced by the generally acknowledged burnout among general practitioners [24], or the presence of office policies not to participate in any surveys [25]. Thus, responses may have been submitted by general practitioners currently interested in learning more about concussion, those who are champions of disseminating emerging concussion best practices, or those purposefully supporting a student project. Further, an incentive for participation offered at the beginning of the recruitment process may increased the initial response rate [26].

In addition, sample bias may have occurred in that there was not a large representation of rural/remote physicians, who may experience different barriers and facilitators to CATT for CME uptake than those in urban settings. As noted by other researchers, further limitations included: method of recruitment (i.e., email may have selected for individuals more comfortable with online and email communication); the short response period may have prevented some physicians from participating; and, other medical professionals who are commonly involved in concussion recognition and treatment (e.g., nurse practitioners and emergency doctors) were not invited to participate [16]. Thus, future researchers should aim to engage with other healthcare practitioners who may seek online concussion education and those who practice in rural/remote settings to better understand their learning needs and insights related to CATT use.

## Conclusion

By surveying physicians in British Columbia, we were able to understand their practices and preferences for receiving CME, barriers and facilitators to implementing the CATT for CME, and provide recommendations to providing concussion education for CME. Barriers to using CATT for CME included lack of time, lack of awareness of the resource and irrelevance to practice. Facilitators included ease of use and succinctness of information. Physician respondents suggested that increasing the awareness of the resource and website, incorporating the CATT into CME events and promoting the CATT on other sites used by physicians may promote uptake of the resource. It is important to understand how physician and healthcare practitioners best engage with online CME in order to reach them in the most efficient and effective manner.

**Summary points**

- Concussion education for medical practitioners is a priority, and continuing medical education (CME) may be a method through which to provide current concussion education.
- The Concussion Awareness Training Tool (CATT) is a free, online, evidence-based, and up-to-date tool that has tailored modules for different audiences, including medical professionals.
- The purpose of this research was to understand physicians’ current CME practices and preferences; understand barriers and facilitators to implementing the CATT as a form of CME; and, to provide recommendations to improve the uptake of the CATT.
- General practitioners were invited to participate in an online survey and follow-up interview via telephone.
- Quantitative data were analyzed using Excel. Interviews were deidentified and transcribed verbatim. Qualitative data, from both the survey and transcribed from the follow-up interviews, were analyzed with NVivo using inductive content analysis.
- Facilitators for use of CATT included: ease of use, accessible, straightforward and that it is concise. Barriers to use and uptake of the CATT included lack of time to complete the module and lack of awareness of the CATT.
- Suggestions to facilitate the uptake of CATT consisted of increasing awareness of the resource and website, incorporating it into CME credits or events, increasing availability of the CATT on other sites used frequently by physicians; and, including information for many different audiences.
- Results from the research can help inform how best to engage medical professionals in CME, as well as how to increase uptake of online learning programs within the medical community.
- Findings will also help inform how to better promote the CATT and recommendations to increase awareness of the resource.
