# Supplementary material for: Barriers and facilitators to the uptake of the Concussion Awareness Training Tool as continuing medical education in primary care
Source: Concussion. 2023 May 19;8(2):CNC106. doi: 10.2217/cnc-2022-0014 (PMC10242435; doi:10.2217/cnc-2022-0014)
Supplement: Supplementary file 1 [file cnc-08-106-s1.docx]

**Supplementary Material: Physician Survey**

| Continuing Medical Education |
| --- |
| 1. How many hours per week do you dedicate to continuing medical education/professional development? |
| 2. When engaging in continuing medical education/professional development what format are you most likely to take part in? |
| 3. What format of continuing medical education/professional development do you most prefer? |
| Concussion Knowledge |
| 4. Please describe your current approach to concussion recognition, diagnosis, treatment and management? |
| 5. How often do you get updated information on concussion recognition, diagnosis, treatment and management? |
| 6. From where do you get updated information on concussion recognition, diagnosis, treatment and management? |
| 7. Are you aware of the Concussion Awareness Training Tool (CATT)?  7a. If yes, where did you learn about the Concussion Awareness Training Tool? |
| 8. Have you previously used the Concussion Awareness Training Tool as a source for updated information on concussion recognition, diagnosis, treatment and management?  8a. If yes, on average, how often do you use the Concussion Awareness Training Tool per month?  8b. If yes, how does the Concussion Awareness Training Tool impact your concussion recognition, diagnosis, treatment and management? |
| 9. If you were previously aware of the Concussion Awareness Training Tool, would you modify it? |
| Concussion Assessment |
| 10. Do you provide care to patients with concussions?  10a. If yes, on average, how many patients with a suspected/diagnosed concussion do you provide care for per month? |
| 11. On average, how long does an evaluation of a patient with a suspected concussion take you (min)? |
| Initial Concussion Assessment |
| 12a. Is a comprehensive history a component of your initial examination of a patient with a suspected concussion?  Please describe. |
| 12b. Is symptom assessment a component of your initial examination of a patient with a suspected concussion?  Please describe. |
| 12c. Is a focused neurological exam a component of your initial examination of a patient with a suspected concussion?  If yes, which of the following is included? (Select all the apply): mental status assessment, cognitive function assessment, coordination assessment, vision assessment, other (if other, please describe) |
| 12d. Is a determination of the clinical status of the patient (including whether there has been improvement or deterioration since time of injury) a component of your initial examination of a patient with a suspected concussion? |
| 12e. Is seeking additional information from parents, coaches, teammates and/or eyewitnesses a component of your initial examination of a patient with a suspected concussion? |
| 12f. Is assessing the need for emergent neuroimaging to exclude a neurosurgically remediable structural brain injury a component of your initial examination of a patient with a suspected concussion? |
| 12g. Are there any other components to your initial examination of a patient with a suspected concussion?  Please describe. |
| Concussion Management |
| 13a. For patients presenting with a diagnosis of a concussion, do you currently recommend nutritional strategies as treatment/management?  Please describe your recommendations within 48 hours of diagnosis vs your recommendations after 48 hours of diagnosis. |
| 13b. For patients presenting with a diagnosis of a concussion, do you currently recommend increased fluid intake as treatment/management  Please describe your recommendations within 48 hours of diagnosis vs your recommendations after 48 hours of diagnosis. |
| 13c. For patients presenting with a diagnosis of a concussion, do you currently recommend sleep strategies as treatment/management?  Please describe your recommendations within 48 hours of diagnosis vs your recommendations after 48 hours of diagnosis. |
| 13d. For patients presenting with a diagnosis of a concussion, do you currently address headaches as treatment/management?  Please describe your recommendations within 48 hours of diagnosis vs your recommendations after 48 hours of diagnosis. |
| 13e. For patients presenting with a diagnosis of a concussion, do you currently recommend appropriate rest as treatment/management?  Please describe your recommendations within 48 hours of diagnosis vs your recommendations after 48 hours of diagnosis. |
| 13f. For patients presenting with a diagnosis of a concussion, do you currently recommend gradual increase in physical activity as treatment/management?  Please describe your recommendations within 48 hours of diagnosis vs your recommendations after 48 hours of diagnosis. |
| 13g. For patients presenting with a diagnosis of a concussion, do you currently recommend gradual increase in cognitive activity as treatment/management?  Please describe your recommendations within 48 hours of diagnosis vs your recommendations after 48 hours of diagnosis. |
| 13h. For patients presenting with a diagnosis of a concussion, do you currently recommend avoidance of activities that put the patient at higher risk for injury until recovery is complete as treatment/management?  Please describe your recommendations within 48 hours of diagnosis vs your recommendations after 48 hours of diagnosis. |
| 13i. For patients presenting with a diagnosis of a concussion, do you currently recommend limitation of screen and device time as treatment/management?  Please describe your recommendations within 48 hours of diagnosis vs your recommendations after 48 hours of diagnosis. |
| 13j. For patients presenting with a diagnosis of a concussion, do you currently recommend Graded Return to School strategies as treatment/management?  Please describe your recommendations within 48 hours of diagnosis vs your recommendations after 48 hours of diagnosis. |
| 13k. For patients presenting with a diagnosis of a concussion, do you currently recommend Graded Return to Sport strategies as treatment/management?  Please describe your recommendations within 48 hours of diagnosis vs your recommendations after 48 hours of diagnosis. |
| 13l. For patients presenting with a diagnosis of a concussion, do you currently offer advice regarding driving as treatment/management?  Please describe your recommendations within 48 hours of diagnosis vs your recommendations after 48 hours of diagnosis. |
| 13m. For patients presenting with a diagnosis of a concussion, do you currently offer advice regarding maintaining social network, connecting with family and friends as treatment/management?  Please describe your recommendations within 48 hours of diagnosis vs your recommendations after 48 hours of diagnosis. |
| 13n. For patients presenting with a diagnosis of a concussion, do you currently recommend any other treatment/management strategies?  Please describe your recommendations within 48 hours of diagnosis vs your recommendations after 48 hours of diagnosis. |
| The Concussion Awareness Training Tool |
| The Concussion Awareness Training Tool was developed as a comprehensive online tool with the aim of enhancing concussion recognition, diagnosis, treatment and management in Canada. The tool consists of five online education modules, each tailored for either medical professionals, coaches, parents, school professionals, or workers and workplaces.  The goal of the Concussion Awareness Training Tool is to “provide information to physicians, nurses, physiotherapists, parents, coaches, athletes, educators and others in a user-friendly, easy-to-understand and easy to navigate format.” The resource is accessible at all times and is free of charge, in addition to providing users flexibility in navigating and reviewing course content. Importantly, the Concussion Awareness Training Tool is updated regularly to incorporate new research and resources. The following is a link to the Concussion Awareness Training Tool: <https://cattonline.com/>  14. What are some factors that might facilitate/encourage you to use the Concussion Awareness Training Tool as a source for continuing medical education on concussion recognition, diagnosis, treatment and management? Please explain how each factor might facilitate/encourage you to use the Concussion Awareness Training Tool.  15. What are some factors that might present as barriers to/discourage you to use the Concussion Awareness Training Tool as a source for continuing medical education on concussion recognition, diagnosis, treatment and management? Please explain how each factor might present as a barrier to/discourage you to use the Concussion Awareness Training Tool.  16. Do you have any additional comments about concussion recognition, diagnosis, treatment and management and/or the Concussion Awareness Training Tool? |
| Demographics |
| 17. What is your occupation? |
| 18. Please indicate the location where you completed/will complete medical school. |
| 19. In what year did you complete/will you complete medical school? |
| 20. Are you currently a family medicine physician/general practitioner? |
| 21. How many years have you been practicing medicine? |
| 22. Are you currently working on a part-time or full-time basis? |
| 23. Do you provide direct patient/clinical care? |
| 24. Please describe the population that you primarily serve. |
| 25. Please describe your primary work setting. |
| 26. How many hours do you usually spend working in an average week (including direct patient care, teaching/education, committee work, administration, research, managing your practice, continuing medical education/professional development)? |
| Follow-up Interview |
| 27. If you are able, we would also be most grateful to have a few minutes of your time to conduct a short follow-up interview. Would you be able to participate in a follow-up interview?  27a. If yes, please provide a phone number at which you can be reached.  27b. If yes, please provide an e-mail at which you can be reached.  27c. Please indicate within the next month, what day and time would be most convenient to reach you |
